# Source-Sink Estimates of Genetic Introgression Show Influence of Hatchery Strays on Wild Chum Salmon Populations in Prince William Sound, Alaska

**DOI:** 10.1371/journal.pone.0081916

**Published:** 2013-12-13

**Authors:** James R. Jasper, Christopher Habicht, Steve Moffitt, Rich Brenner, Jennifer Marsh, Bert Lewis, Elisabeth Creelman Fox, Zac Grauvogel, Serena D. Rogers Olive, W. Stewart Grant

**Affiliations:** 1 Commercial Fisheries Division, Alaska Department of Fish and Game, Anchorage, Alaska, United States of America; 2 Commercial Fisheries Division, Alaska Department of Fish and Game, Cordova, Alaska, United States of America; 3 School of Fisheries and Ocean Sciences, University of Alaska Fairbanks, Juneau, Alaska, United States of America; Ecole Normale Supérieure de Lyon, France

## Abstract

The extent to which stray, hatchery-reared salmon affect wild populations is much debated. Although experiments show that artificial breeding and culture influence the genetics of hatchery salmon, little is known about the interaction between hatchery and wild salmon in a natural setting. Here, we estimated historical and contemporary genetic population structures of chum salmon (*Oncorhynchus keta*) in Prince William Sound (PWS), Alaska, with 135 single nucleotide polymorphism (SNP) markers. Historical population structure was inferred from the analysis of DNA from fish scales, which had been archived since the late 1960’s for several populations in PWS. Parallel analyses with microsatellites and a test based on Hardy-Weinberg proportions showed that about 50% of the fish-scale DNA was cross-contaminated with DNA from other fish. These samples were removed from the analysis. We used a novel application of the classical source-sink model to compare SNP allele frequencies in these archived fish-scales (1964–1982) with frequencies in contemporary samples (2008–2010) and found a temporal shift toward hatchery allele frequencies in some wild populations. Other populations showed markedly less introgression, despite moderate amounts of hatchery straying. The extent of introgression may reflect similarities in spawning time and life-history traits between hatchery and wild fish, or the degree that hybrids return to a natal spawning area. The source-sink model is a powerful means of detecting low levels of introgression over several generations.

## Introduction

Interactions between hatchery-reared and wild Pacific salmon can be a source of genetic change within and among wild populations [1]. Even when the initial hatchery brood stock is drawn from nearby wild populations, hatchery culture can change the genetic makeup of the hatchery population, especially in segregated hatchery populations, in which brood stocks are selected from fish returning to the hatchery [2], [3]. Some hatchery fish inevitably stray into wild populations, and the degree of influence these fish have on wild populations is related to the intensity of stock enhancements [4], the amount of genetic divergence between hatchery and wild populations [4], [5], and the extent of genetic introgression of hatchery genotypes into wild populations [6]. A key variable moderating the effects of hatchery-reared strays on wild populations appears to be the degree of life-history divergence between the hatchery and wild populations [4], [6], [7].

The effects of stray hatchery fish on wild populations can be measured in several ways. Fin clips, physical tags, and thermally marked otoliths allow for the direct detection of hatchery fish in natural spawning areas [8]. While stray hatchery fish may influence wild populations ecologically [9], they may not necessarily mate with wild fish. Even matings between hatchery and wild fish may not lead to introgressive hybridization, because hybrid offspring may be less fit than pure wild fish and may not successfully compete for mates or survive to spawn [3], [10]. Long-term monitoring of life-history traits can sometimes demonstrate the effects of hatchery strays on wild populations. For example, run timing in a wild population of coho salmon (*Oncorhynchus kisutch*) shifted to earlier times as a result of the genetic influence of strays from a hatchery in which eggs were taken from the early portion of the run [11].

One approach to estimating genetic introgression in salmonids is based on individual-based methods that attempt to identify hatchery-wild hybrid and backcross fish [6], [12]–[14]. These methods use genotypes in a contemporary sample and Bayesian probabilities to estimate hybridizations with fit to Hardy-Weinberg proportions and linkage disequilibria among genotypes. Here, we use an alternative approach by comparing DNA in archived fish scales with DNA in contemporary samples to detect possible allele-frequency shifts in wild populations influenced by hatchery strays. Historical samples have previously been used to estimate the genetic structure of historical populations [15], [16] and genetic shifts from hatchery supplementation [10], [15], [17]. We developed a novel application of the classical source-sink model to track allele-frequency changes in wild populations due to introgression from stray hatchery-reared fish.

**Figure 1:**
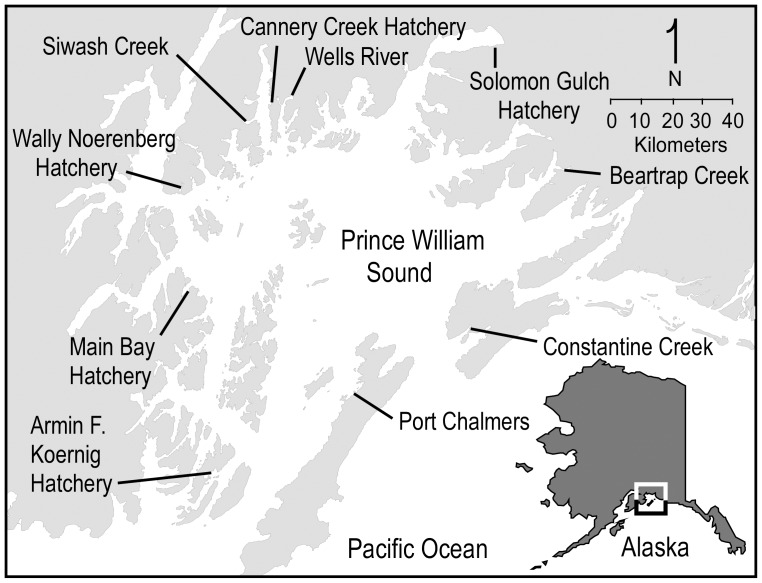
Locations of chum salmon hatcheries and release sites and sampled natural-spawning sites in Prince William Sound, Alaska.

Our study focuses on the genetic influence of hatchery strays on chum salmon (*Oncorhynchus keta*) populations in Prince William Sound (PWS), Alaska (Figure 1). Over 200 streams in PWS support wild spawning aggregations, with an estimated ten-year (2001–2010) annual average of 273,100 fish migrating to index spawning areas. The total number of wild spawners in PWS is unknown, but is thought to be much larger than abundances in these index streams. The Prince William Sound Aquaculture Corporation (PWSAC) released about 139.5 million chum salmon fry in 2011 [18], and the total return to PWS of hatchery and naturally spawned fish averaged about 4.2 million fish annually over the last ten years [19]. Since 1976, chum salmon have been released in PWS at several sites, including Armin F. Koernig (AFKH), Main Bay (MBH), Wally Noerenberg (WNH), Cannery Creek (CCH), and Solomon Gulch (SGH) hatcheries, and Port Chalmers on Montague Island (Table 1). Although these five hatcheries produced chum salmon between 1985–1994, chum salmon culture was suspended, except at WNH. Hence, WNH has been the chief source of hatchery releases for about two decades. Wells River was the largest source of hatchery brood stock for this hatchery, but a small number of fish came from Beartrap Creek until 1986, when the number returning to WNH was large enough to meet production goals.

**Table 1:** Chum salmon production in Prince William Sound, Alaska hatcheries.

Hatchery	Duration	Brood source	Maximum number ofreleases (millions)	Maximum number ofreturns (thousands)
**Wally Noerenberg**	1983–present	Wells River, Beartrap Creek^a^	165^b^	5000
**Armin F. Koernig**	1977–1985	Larson Creek, Sunny Creek, Fidalgo Creek	34	420
**Cannery Creek**	1978–1989	Wells River, Siwash Creek, Eagle River	4	36
**Main Bay**	1982–1987	Wells River	38	321
**Solomon Gulch**	1981–1994	Crooked Creek, Spring Creek, Fidalgo Creek	17	270

^a^ Last used as brood stock in 1986.

^b^ Includes releases at Armin F. Koernig Hatchery and Port Chalmers remote release site.

Our study has three components. First, we develop a novel method for quality control of single-nucleotide polymorphism (SNP) genotypes. This QC was critical because the analysis relied on DNA extracted from archived scales originally sampled decades ago to age fish. The handling of fish during sampling promotes cross-contamination between scales, which can affect genotyping. Accurate genotypes are especially important for detecting low levels of introgression. Second, we compare the historical genetic population structure estimated from archived scale samples to contemporary population structure. If introgression from hatchery populations into wild populations is occurring, wild populations are expected to gradually resemble hatchery populations. Third, we develop a novel application of the classic source-sink model and use SNP frequencies in historical and contemporary samples to quantify introgression rates of hatchery chum salmon in four naturally spawning populations.

## Materials and Methods

### Ethics Statement

All work was conducted in accordance with animal welfare guidelines stipulated in field collection permit CF-2009-0019, issued by the State of Alaska Department of Fish and Game (ADF&G), Juneau, AK. This study did not involve endangered or protected species and the sampling locations were not privately-owned or protected in any way.

### Archive Sample Selection and Field Collection

We sampled chum salmon from WNH and from four streams in PWS (Beartrap Creek, Constantine Creek, Siwash Creek, and Wells River; Table 2, Figure 1), for which historical scale samples were also available. These populations differ in spawning time and span a range of distances from hatchery release sites. We chose these populations, in part, because at least 200 archived scales were available from years before hatchery operations began. In 2008, 2009, and 2010, the axillary processes of 500 fish at each location were collected, placed into individual vials with 95% ethanol, and stored at room temperature. Otoliths were also dissected from these fish to search for thermal marks indicating hatchery culture. Otolith markings in the contemporary field collections, indicating hatchery-origin strays, were removed from the analysis. A target sample size of 200 wild fish without thermally marked otoliths was selected from the 500 fish from each stream and used for genetic analysis (Table 2). In addition, axillary processes were collected from 200 freshly killed brood stock at the WNH in 2008 and 2009. Samples from wild populations were pooled into 8 collections based on geographic location (Siwash Creek, Wells River, Beartrap Creek, and Constantine Creek) and time of collection (historical 1964–1982, and contemporary 2008–2010). Samples from the WNH, collected 2008–2009, were pooled into a single collection for analysis. After quality control (see below), sample sizes for the historical DNA samples ranged from 50–70 and for contemporary samples from 193–585.

**Table 2:** Collection information for samples of chum salmon from Prince William Sound, Alaska, used to examine hatchery-wild genetic introgression.

					Tests for contamination			
Location (year)	Abbrev^a^	Initial *N*	Identified hatchery strays	20% missing loci	Microsatellites	Hardy-Weinberg	Final *N*	Proportion of strays (±SD)^b^
**Wally Noerenberg Hatchery (2008–2009)**	WNH	200	3	4	0	0	193	NA^d^
**Siwash Creek (1964–1982)**	SC-H	121	NA^d^	6	54	9	52	NA
**Siwash Creek (2008–2010)**	SC-C	311	4	5	0	4	298	0.250^e^ ±0.046
**Wells River** c **(1964–1982)**	WR-H	200	NA	52	81	17	50	NA
**Wells River (2008–2010)**	WR-C	600	9	3	0	8	580	0.038^e^ ±0.015
**Beartrap Creek** c **(1964–1982)**	BC-H	201	NA	13	103	14	70	NA
**Beartrap Creek (2008–2010)**	BC-C	600	1	16	0	12	571	0.002^f^ ±0.005
**Constantine Creek (1964–1982)**	CC-H	200	NA	11	101	21	67	NA
**Constantine Creek (2008–2010)**	CC-C	600	1	5	0	9	585	0.005^f^ ±0.007
**Total**		3033	18	115	339	94	2466	

*N*), and documented hatchery-wild straying rates for contemporary spawning areas. Table includes results of hatchery strays identified through otolith marks, sample quality control and contamination tests (see text for description), initial and final sample sizes (

^a^ Historical (-H) and contemporary (-C) collections.

[8].^b^ From

^c^ Source of hatchery brood stock.

^d^ Not applicable.

^e^ Mean from 2004 to 2010.

^f^ Mean from 2005 to 2010.

### Laboratory Analysis

Genomic DNA was extracted using a DNeasy® 96 Tissue Kit by QIAGEN® (Valencia, CA). Each fish was screened for 185 nuclear DNA and 3 mitochondrial DNA SNP markers (Table S1) [20]–[25]. SNP assay reactions were conducted in two BioMark 96.96 Dynamic Arrays (Fluidigm). Reaction cocktails (7.2 nL) consisted of 1×TaqMan Universal Buffer (Applied Biosystems), 1.5 U AmpliTaq Gold DNA Polymerase (Applied Biosystems), 9 mM of each polymerase chain reaction (PCR) primer, 2 mM of each probe, 1×DNA Assay Loading Buffer (Fluidigm), 12.5×ROX (Invitrogen), and 0.01% Tween-20. PCR amplification was performed with a BioMark IFC Cycler with an initial denaturation period of 10 min at 95°C followed by 50 cycles of 92°C for 15 s, and one step at 60°C for 1 min. Dynamic Arrays were read with a BioMark Real-Time PCR System after amplification and scored visually with BioMark Genotyping Analysis software (Fluidigm).

### Quality Control

Several steps were taken to ensure genotype accuracy. First, 8% of the samples in each collection were reanalyzed for each marker to ensure reproducibility and to identify possible laboratory handling errors. We assumed that any inconsistency was due equally to genotyping errors in the initial analysis and to errors during quality control. Second, we excluded samples with poor quality DNA, in which individuals lacked scores for more than 20% of the SNP markers [25]. This reduced the chance that the remaining loci were also mis-genotyped, as poor quality DNA provides less replicable genotypes across loci than high quality DNA. Third, we eliminated SNP markers that were genotyped for fewer than 55 fish in an archived scale sample. Fourth, we tested genotypic frequencies for each locus in each collection for fit to Hardy-Weinberg expectations (HWE) with Fisher’s exact test [26], as implemented in GENEPOP 4.0.10 [27]. Critical values (α = 0.01) were adjusted for multiple tests within collections and multiple tests across markers within a collection [28]. A locus was removed from the analyses if it deviated from HWE in a majority of the collections. Fifth, we removed markers that were invariant in all collections. Sixth, to ensure that the analyses were based on independent markers, we tested all pairs of markers for linkage disequilibrium within each collection using GENEPOP with 100 batches of 5,000 iterations. We assumed that pairs of loci were linked if they exhibited significant (*P*<0.05) linkage disequilibrium in more than half of the collections, or if the markers were known to be linked. When we found linkage between a pair of loci, the locus with the lower heterozygosity, or the locus with the larger percentage of missing data in the historical samples was discarded.

### Contaminated Samples

We anticipated that DNA extracted from the archived scales might be contaminated with DNA from other scales, because the archived scales had been used for aging and had not been collected with protocols specifically for DNA analysis. DNA is located chiefly on the surface of a scale in epithelial tissue and mucous. We devised two methods to detect fish-to-fish contamination. First, we analyzed the archived fish DNA for 7 microsatellites [29], Oke4, Oke11 [30], Oki1L, Oki1U [31], Ots2.1L, Ots2.1U [32], Ots103 [33] (Table S2). Contaminated samples were expected to display more than two alleles for at least one locus. Fish with genotypes showing more than two alleles were excluded from further analysis.

Second, we used model selection based on genotypic frequency expectations. This method makes three key assumptions: 1) loci are in HWE and linkage equilibrium, 2) contaminated individuals are contaminated by one, and only one, other fish from the same population, and 3) if a fish is contaminated at one locus, it is contaminated for all other loci. Genotypes were subscripted by collection 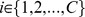, individual 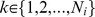, and locus 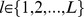 and were represented by the unit vector
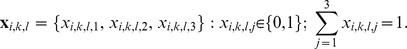



This data vector takes three possible states corresponding to the genotypes *AA, Aa*, and *aa.* We modeled 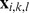 as
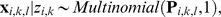
Where 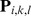 is a vector of genotype score probabilities with components



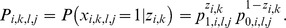



Here, 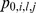 and 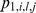 are the uncontaminated and contaminated genotypic frequencies, respectively, of genotype index 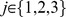 at locus *l*, and are simple functions of the allele frequencies, 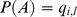 (Table 3), while 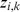 is a Bernoulli random variable equal to one if individual 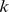 is contaminated, and to zero if the individual is uncontaminated. We placed a Rannala-Mountain [34] prior on the allele frequencies and a Bernoulli prior on 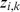, with probability of success equal to 0.5. The variable 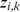 was sampled for each individual from its posterior distribution to determine which of the two models fit individual 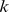 the best. We ran two Markov Chain Monte Carlo (MCMC) chains in OpenBUGS (Table S3) for 100,000 iterations, discarding the initial 50,000 iterations from each chain as burn-in. To initialize the two chains, individuals were set as contaminated in one chain and uncontaminated in the second chain, and convergence between the chains was assessed by examining trace plots of the two chains. Since the prior for 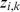 gives equal weight to each model, its posterior mean can be viewed as the posterior probability that scale extraction *k* is contaminated, given only two possible models. Individuals with posterior probabilities greater than 75% corresponded to Bayes factors greater than 3 [35], so were excluded.

**Table 3:** Competing models to detect DNA contamination among individuals (*k)* within a collection (*i*) across loci (*l*).

		Uncontaminated model (*z_i_*_,*k*_ = 0)		Contaminated model (*z_i_*_,*k*_ = 1)	
Genotype index (*j*)	Apparent genotype	Probability	True genotype	Probability	True genotype
1	AA	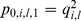	*AA*	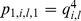	*AAAA*
2	Aa	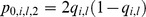	*Aa*	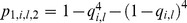	*Aaaa*, *AAaa*, *AAAa*
3	aa	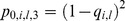	*aa*	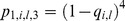	*aaaa*

Genotype index is a value assigned the apparent genotype observed during allele scoring. In uncontaminated and contaminated individuals, the probability of observing these apparent genotypes is estimated by Hardy-Weinberg expectations based on a single individual and on two individuals, respectively. See text for description.

A total of 115 fish across samples could not be genotyped at 20% or more loci and were removed from further analysis (Table 2). Thirty-two SNP loci were removed because of low amplification success in historical scale samples. Locus *Oke_U1016-154* departed from HWE in the historical collections and was removed from subsequent analysis. Additionally, the three mitochondrial DNA (*Oke_Cr30*, *Oke_Cr386*, *Oke_ND3-69*) and two nuclear (*Oke_U1010-154*, and *Oke_U202*) loci were removed, because they were invariant in our samples. Fourteen pairs and one triplet of loci showed significant linkage disequilibrium in more than 50% of the collections (Table S4). One exception was the pair *Oke_psmd9188* and *Oke_psmd957*, which showed linkage disequilibrium in 4 of the 9 collections. However, this pair is known to be linked. Hence, these 16 loci were discarded. A list of SNP heterozygosities appears in Table S1. After these analyses, genotypes for 135 SNP loci were used to detect genetic introgression of the WNH stock into wild populations. We removed 339 fish from the archived scale collections, because microsatellite genotypes indicated that DNA extractions were contaminated. Finally, we removed 94 contemporary and historical individuals with Bayes factors indicating contamination (Table 2).

### Population Analysis

The four contemporary samples were large enough (*N* = 298 to 585) to subdivide and test for differences among the three collection years, 2008–2010. Fisher’s exact test for 12 comparisons detected a significant difference (*P* = 0.017) between Wells River 2008 and 2009, but not between collection years at the other sites. The historical samples were too small to test for temporal differences. Otolith markings indicated that 18 fish from contemporary field collections were hatchery-origin strays and were removed from the analysis.

For the SNP data, we estimated observed (*H*_o_) and expected (*H*_e_) heterozygosities averaged over loci with ARLEQUIN 3.5.1.2 [36]. Differentiation between populations was estimated in four ways. ARLEQUIN was used to estimate components of variability among samples, including *F*_ST_ (differentiation among spawning sites), *F*_SC_ (differences between temporal samples within sites), and *F*_IS_ (mean variability among individuals within collections). Second, pairwise exact tests [37], [38] were made between all samples with GENEPOP with 5000 burn-in steps, and 1000 batches of 1000 Markov Chain steps per batch. Third, pairwise *F*_ST_ values were calculated with the R package ‘hierfstat’ [39]. The R package ‘ape’ [40] was used to produce 1000 bootstrap neighbor-joining (NJ) trees from pairwise *F*_ST_ values and a consensus tree. Fourth, we used STRUCTURE 2.3.3 [12] to estimate population structure from individual assignments. Genotypic data in the collections were pooled and tested with the ‘admixture’ model using sample location and date (historical or contemporary) with 50,000 MCMC steps, following a burn-in of 10,000 steps. Genotypic data were tested with *K* = 1–9 populations.

### Source-sink Model of Genetic Introgression

**Figure 2:**
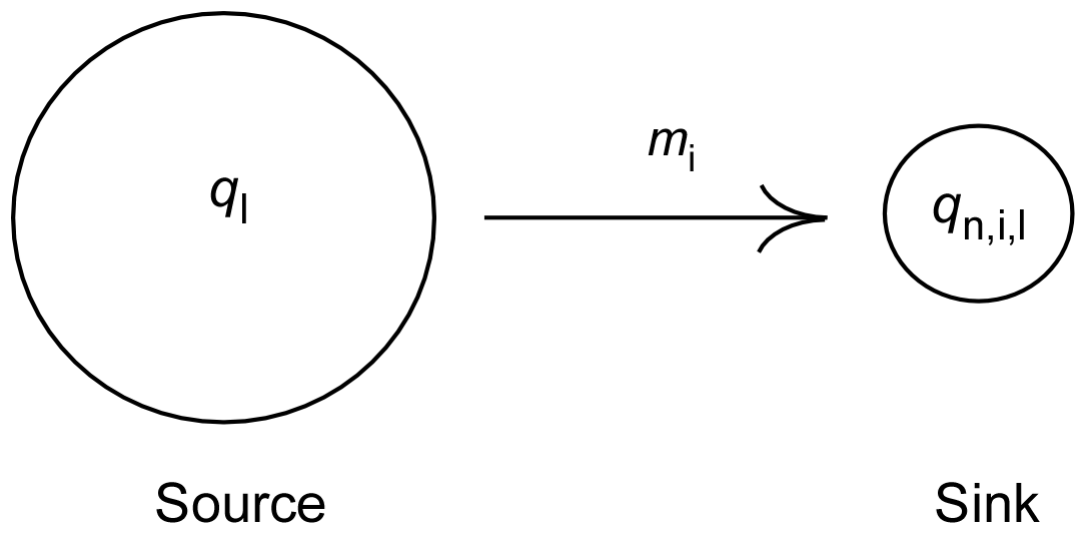
Diagram of a model of genetic introgression based on the classic source-sink model of migration. Explanation of variables: *q_l_* is the allele frequency at a locus in a source population and is assumed to be unchanging over *n* generations of introgression. *q_n,i,l_* is the allele frequency at locus, *l,* in a wild sink population, *i* after *n* generations.



We used the source-sink model [41] to develop a way of estimating genetic introgression (Figure 2). In our model, we expected allele frequencies in wild sink populations to shift as hatchery strays from the source population bred with wild fish. Allele frequencies at a locus in a sink population, 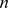 generations after the onset of introgression, are

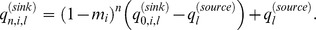



Here, *m_i_* is the rate of introgression into population *i*. The same equation with the same rate of introgression applies to all loci individually. In this model, 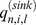 is not treated as a free parameter, but as a function of the free parameters 
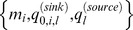
. Without introgression *m_i_ = *0, so that after *n* generations of introgression
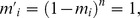
and



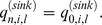



With introgression 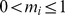, so that
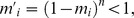
and



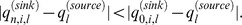



Therefore, the slope of the plot of 
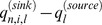
 against across loci indicates the effect of introgression. In the absence of introgression, these points are expected to fall along the replacement line *y* = *x*, so the slope of the regression is 1.0. With introgression, these points are expected to fall on a line with a slope of less than 1.0. The model was run in OpenBUGS (Table S5). We placed Rannala-Mountain priors [34] on 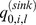 and 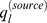 and a flat normal prior on each 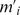 with a mean of zero and a variance of one-thousand. We then ran two chains with disparate starting values for 100,000 iterations, discarding the first 50,000 iterations as burn-in. The posterior mean and 95% credible intervals were estimated for each 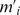.

## Results

### Population Analysis

Overall, 1.45% (*F*_ST = _0.0145, *P*<0.00001) of the total variability was due to differences among the four spawning sites, and 0.15% (*F*_SC_ = 0.0015, *P*<0.0001) was due to temporal differences between samples at the same site. Fisher’s pairwise exact tests for genetic differentiation echoed these results, showing significant differences (*P*<0.001) between historical and contemporary collections from the same sites (Table 4). The remaining 98.40% of the variability was due to genotypic differences among individuals within samples (*F*_IS_). The amount of differentiation among the four historical samples was slightly larger (*F*_ST_ = 0.0161, Fisher’s exact test *P*<0.001) than among the four contemporary samples (*F*_ST_ = 0.0158, Fisher’s exact test *P*<0.001).

**Table 4:** Estimates of genetic diversity and divergence (*F*_ST_) between historical (H) and contemporary (C) samples of chum salmon from Prince William Sound, Alaska.

	WNH	SC-H	SC-C	WR-H	WR-C	BC-H	BC-C	CC-H	CC-C
**WNH**	**0.3319**	–	***	–	***	–	***	–	***
**SC-H**	0.0062	**0.3393**	***	***	–	***	–	***	–
**SC-C**	0.0049	0.0018	**0.3277**	–	***	–	***	–	***
**WR-H**	0.0029	0.0095	0.0036	**0.3445**	***	***	–	***	–
**WR-C**	0.0008	0.0022	0.0031	0.0012	**0.3211**	–	***	–	***
**BC-H**	0.0041	0.0111	0.0040	0.0060	0.0017	**0.3251**	***	***	–
**BC-C**	0.0028	0.0027	0.0041	0.0017	0.0029	0.0011	**0.3279**	–	***
**CC-H**	0.0097	0.0161	0.0069	0.0138	0.0044	0.0143	0.0049	**0.3224**	***
**CC-C**	0.0100	0.0049	0.0107	0.0042	0.0121	0.0051	0.0131	0.0009	**0.3099**

Table 2 for sample abbreviation. *F*_ST_ (below diagonal), expected heterozygosity *H*_e_ (diagonal in **bold**), and Probability of Fisher’s exact test over loci for selected comparisons (above diagonal) between historical (H) and contemporary (C) collections. See

*P*<0.001.

**Figure 3:**
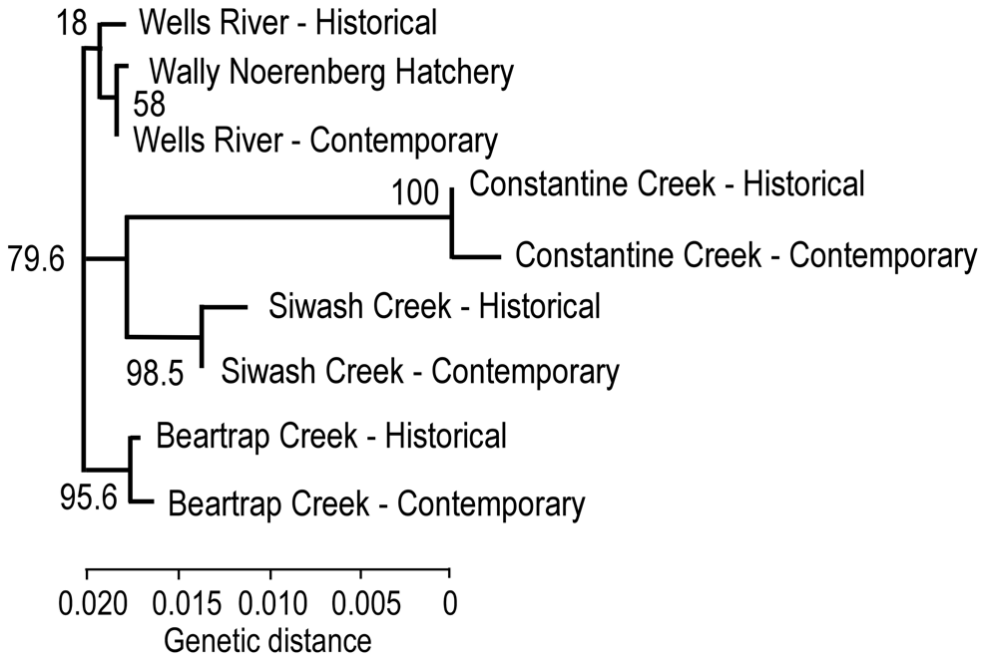
Neighbor-joining tree of *F*_ST_ between chum salmon samples from Prince William Sound, Alaska. Numbers in the tree represent bootstrap support for a node.

A consensus NJ tree of *F*_ST_ showed that the historical and contemporary collections from Wells River were most similar to the WNH stock. Each of the population pairs, except Wells River, had high bootstrap support in the tree (Figure 3). In three of the pairs of temporal and contemporary samples, *H*_o_ and *H*_e_ were marginally smaller in contemporary samples than in historical samples (Table 4).

**Figure 4:**
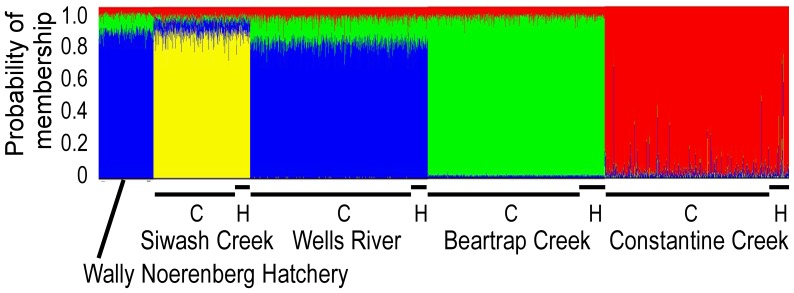
STRUCTURE analysis of genotypes at 135 nuclear SNPs in chum salmon from Prince William Sound, Alaska. Individual assignments for contemporary (C) and historical (H) collections with *K* = 4.

STRUCTURE indicated that the 9 collections (both historical and contemporary) best fit a four-population model (Figure 4). Generally, the results showed genetic differentiation among the four populations that we sampled. Most individuals were assigned back to their population with probabilities of 85–95%. No differences in the probabilities of assigment appeared between archived and contemporary genotypes from a particular location. However, small genetic components from other populations appeared in each population. A small genetic signal (red) from Constantine Creek appeared in WNH and the three other populations. A small signal (green) from Beartrap Creek appeared in WNH and Wells River, but was absent in Siwash and Constantine creeks. A small signal (blue) from Wells River appeared in fish from Constantine and Siwash creeks, but a large Wells River signal appeared in WNH, reflecting the origins of WNH fish from Wells River. Virtually no genetic signal (yellow) of Siwash Creek fish appeared in WNH and the other populations. It is uncertain whether these extrinsic components are due to gene flow and hybridization, or to the similarity of some SNP genotypes among populations.

### Estimates of Genetic Introgression

**Figure 5:**
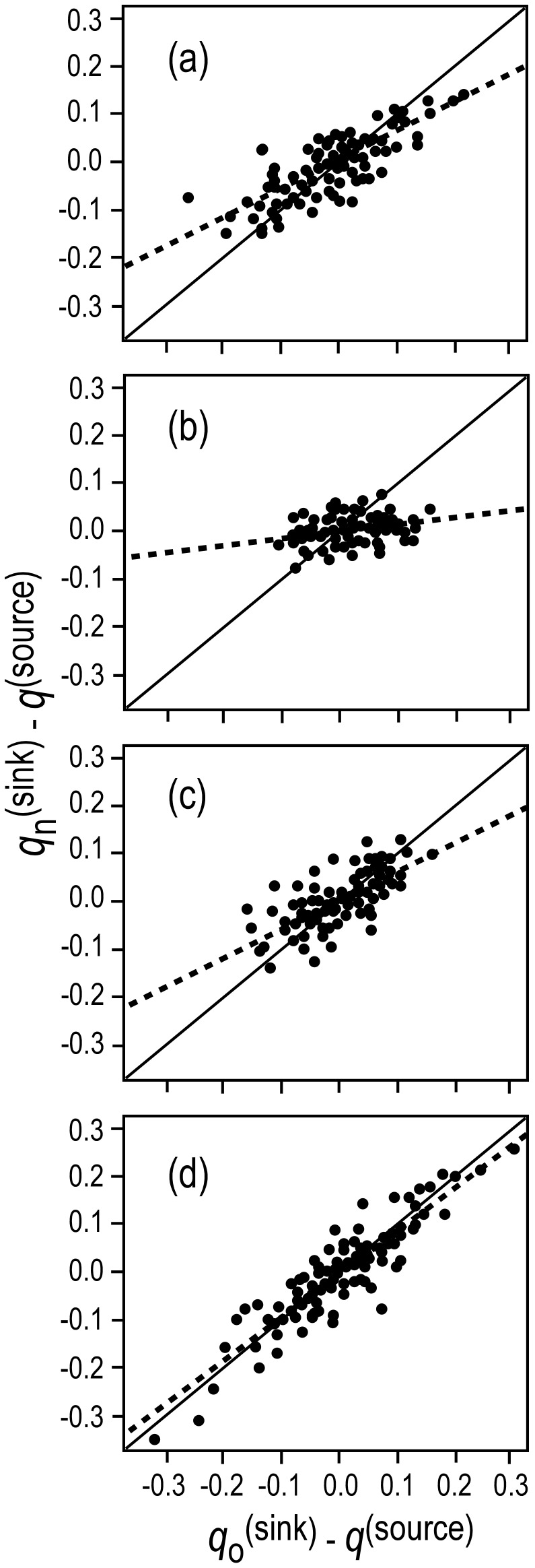
Plots of 
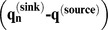
 versus 
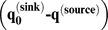
 for 135 SNP loci in chum salmon in Prince William Sound, Alaska. Dashed line represents observed curve and solid line represents expected curve without introgression. (a) Siwash Creek, (b) Wells River (c) Beartrap Creek, (d) Constantine Creek.

**Figure 6:**
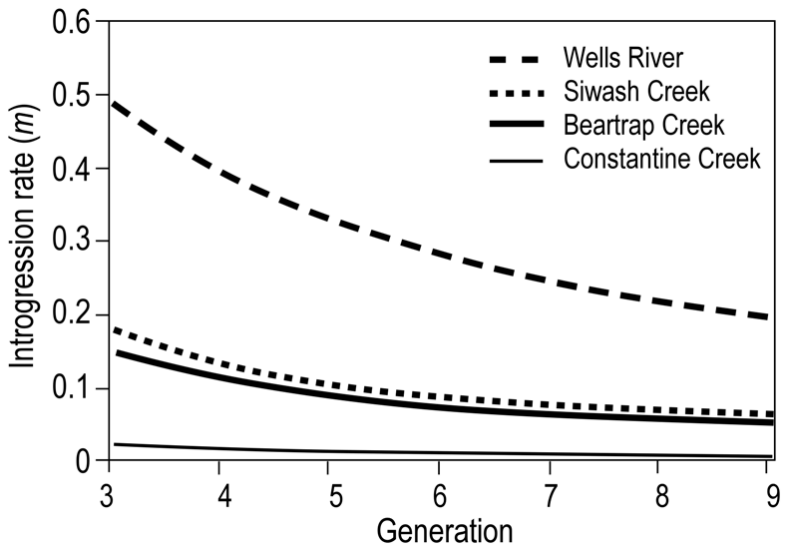
Approach to equilibrium of per-generation introgression coefficients, *m*, in natural chum salmon spawning areas in Prince William Sound, Alaska.

In our source-sink model, evidence for introgression appeared as a convergence with time between allele frequencies in a wild population with allele frequencies in the hatchery. This convergence produced a positive deviation from the expected one-to-one relationship between the slope of the difference between source and sink allele frequencies 
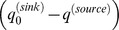
 before hatchery production and about six generations later
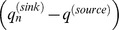
. Three sample pairs for Siwash Creek, Wells River, and Beartrap Creek showed a shift in allele frequencies, with the strongest shift appearing in Wells River (Figure 5a,b,c). Less introgression was detected in Constantine Creek (Figure 5d). Bayesian estimates of the per-generation introgression rate (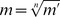, where *n = *6 generations) from the source-sink equation indicated that 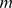 was significantly larger than zero in each of the four populations (Table 5, Figure 6). Wells River showed the largest rate of introgression (*m* = 0.257, 95% PD: 0.209–0.328), and Siwash and Beartrap creeks showed intermediate levels of introgression (*m = *0.066, 0.052–0.081 and 0.060, 0.046–0.074, respectively). Constantine Creek showed the lowest, but still significant, level of introgression (*m = *0.011, 0.004–0.017).

**Table 5:** Source-sink model estimates of genetic introgression of hatchery genes into wild populations of chum salmon from Prince William Sound, Alaska.

Location	*m*	2.5%	97.5%
**Siwash Creek**	0.066	0.052	0.081
**Wells River**	0.257	0.209	0.328
**Beartrap Creek**	0.060	0.046	0.074
**Constantine Creek**	0.011	0.004	0.017

Introgression rate 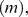 and Bayesian 95% credibility interval in contemporary populations.

## Discussion

Our analysis of archived scales and contemporary collections of chum salmon in Prince William Sound demonstrates genetic introgression from hatchery strays into the four natural spawning populations examined in this study. The use of the source-sink model appears to have provided more power for detecting introgression than the use of individual assignments with STRUCTURE, which has commonly been used in other studies to detect introgression. Both STRUCTURE and NEWHYBRID [13] attempt to identify F_1_ and F_2_ hybrids and backcrosses in a population, but may not detect the effects of introgression over several generations. Additionally, our application of the sink-source model to analyze DNA in archived scales and contemporary samples allowed us not only to detect allele-frequency shifts, but also to estimate introgression rates over 6–7 generations.

We expended considerable effort on quality control to increase the accuracy of genotypes so that our analyses had the power to detect small hatchery influences on wild populations. First, we implemented methods to minimize the potential for genotyping bias in these procedures. For example, because some loci may be inherently more scorable than other loci in samples with poor quality DNA, we excluded the latter samples to reduce the inclusion of suspect genotypes. Poor quality DNA can yield valid genotypes for some loci, but not others.

Second, we devised two methods to screen for DNA contamination between scale samples, including the supplementary use of microsatellites, and model selection based on Hardy-Weinberg Expectations (HWE) for SNP loci. The estimation of genotype and allele frequencies critically depends on excluding contaminated samples. Inferences of introgression, in turn, depend on accurate estimates of these frequencies. Therefore, we excluded any archival or contemporary samples that appeared to be contaminated with DNA from other fish. About 1.2% (0–2%), on average, of the DNA extractions from the five contemporary samples tested positive for contamination with the HWE-based test, but not with microsatellites (Table 2). These putative contaminations appear to be false positives. On the other hand, 55.2% (49–61%), on average, of the extractions from the four archived scale samples tested positive for contamination, much higher than the false-positive rate for contemporary samples. These stringent measures of quality control provide considerable confidence in the subsequent analyses.

### Shifts in Genetic Diversity and Population Structure

Persistent straying from a single-source population over several decades can potentially erode genetic diversity among populations. Previous studies of genetic population structure in chum salmon in PWS showed a considerable amount of genetic diversity among populations in the 1990s [42], [43]. These studies detected several statistically significant partitions between populations on the eastern side of PWS, and a major partition between eastern and western PWS populations. Our sampling was not geographically extensive enough to revisit the east-west partition; nevertheless, the STRUCTURE analysis showed strong geographic structure among populations around PWS. A small allele-frequency shift among contemporary samples (*F*_ST_ = 0.0158), relative to the historical samples (*F*_ST_ = 0.0161) may indicate convergence among these wild populations because of a common source of migrants. A similar temporal shift was detected among populations of Atlantic salmon (*Salmo salar*) in France; divergence at 17 microsatellite loci among historical samples was larger (mean *F*_ST_ = 0.080, range 0.057–0.096) than among contemporary samples (mean *F*_ST_ = 0.04, range 0.013–0.071) [44]. An allele-frequency shift and drop in heterozygosity in some PWS chum salmon populations are predictions of the Ryman-Laikre effect of hatchery strays on wild populations [45]. However, we cannot exclude the possibility that a decadal increase in the amount of gene flow between wild populations in PWS in response to an environmental regime shift [46] may also have led to greater similarity among wild populations.

### Source-sink Model of Genetic Introgression

Our genetic analysis also indicates that the most recent contributions of brood stock to the WNH from Wells River swamped the genetic signatures of previous brood stock from other localities. Hence, we used genotypes in the WNH sample as the ‘source’ in our source-sink estimations of introgression. Even though the absolute amount of differentiation was small (*F*_ST_ = 0.001 to 0.010), the exact tests of differentiation were significant for each hatchery-sample comparison because of the power provided by a large number of markers. Hence, the individual assignments made with STRUCTURE did not indicate a substantial amount of recent hybridization between hatchery brood stock and wild populations, even though our source-sink model showed significant allele-frequency shifts after six generations of hatchery supplementation.

### Genetic Imprints of Hatchery Strays on Wild Populations

The results for the four populations that we sampled illustrate several bio-complexities of hatchery influences on wild populations. The straying of hatchery fish is well documented by the presence of fish with thermally marked otoliths in streams where wild fish spawn. Brenner et al. [8] sampled chum salmon from 2004 to 2010 in the four streams used in our study and found that 0.0 to 62.6% of the fish were of hatchery origin (Table 6). Beartrap Creek had the lowest percentage of stray hatchery fish, with a four-year average of 0.2%, whereas Siwash Creek had a five-year average of 25.1%. Wells River, Beartrap, and Constantine creeks support the three largest chum salmon spawning populations in PWS, with average escapements that are an order of magnitude larger than those of Siwash Creek, which ranks 24^th^ in size. The abundances and locations of populations relative to hatcheries provide a background for understanding the biological implications of our genetic introgression estimates.

**Table 6:** Percentage stray hatchery-reared chum salmon found in naturally spawning populations of chum salmon in Prince William Sound, Alaska from 2004 to 2010.

Population	Year								
	2004	2005	2006	2007	2008	2009	2010	Mean	Mean population census size
**Siwash Cr.**	35.9	62.6	8.7	7.9	5.1	38.7	6.7	25.1	3,000
**Wells R.**	2.1	2.6	6.3	3.2	2.2	7.4	3.1	3.8	23,100
**Beartrap Cr.**	ND^a^	0.0	0.0	0.4	0.4	0.0	0.0	0.2	22,700
**Constantine R.**	ND	0.5	0.5	0.0	0.2	0.8	1.2	0.5	15,600

a No data.

Data from [8].

#### Constantine creek

The sample from Constantine Creek had the second smallest proportion of stray hatchery chum salmon (0.5%, 2005–2010) and the lowest level of genetic introgression (Figure 6). This is the third largest chum salmon producer in PWS and is located farthest from any hatchery chum salmon release site of the streams considered here. Notably, the peak run timing of Constantine Creek is later than that for fish returning to WNH by about 16 days (ADF&G unpublished data). Thus, the low introgression rate may be due to a combination of a low proportion of stray hatchery fish in the population, a large geographic distance from hatchery release sites, a difference from hatcheries in run timing, and a large population size that resists introgression.

#### Siwash creek

A high level of genetic introgression was expected in this small population (mean 3000 adults), because it is located close to the WNH and because it receives a large number of hatchery strays [8]. However, the source-sink model detected only a small amount of introgression. The low incidence of introgression may variously be due to a mismatch between the run timings of hatchery and local wild fish, behavioral differences between hatchery and wild fish that reduce successful inter-breeding, low survivals of hybrid offspring, and poor homing of hybrids to Siwash Creek. Perhaps the most important factor is the contrast in run timing between WNH and Siwash Creek fish that reduces the chances of mating between hatchery and natural fish. Aerial surveys in PWS indicate that the median time of return to Siwash Creek lags the return times of WNH Wells-River fish by about 31 days (ADF&G unpublished data). The earlier run timing of hatchery fish leads to a greater number of strays early in the season [8] when local wild fish are not spawning.

Unexpectedly low levels of introgression, despite persistent straying, have been found in other salmonids. Hendry et al. [47] showed that populations of sockeye salmon (*Oncorhynchus nerka*) could exchange large numbers of migrants each generation, yet remain genetically distinctive because of reduced reproductive success. In addition to straying intensity, introgression may be influenced by local population size [17] and by the survival and reproductive success of hatchery fish in the wild [3], [5], [48]. The use of hatchery brood stock with divergent run timing may, in fact, reduce hybridization between hatchery strays and wild fish in this system [49].

#### Beartrap creek

This is the second largest chum salmon run in PWS (ADF&G unpublished data). Wild populations in this creek showed intermediate levels of genetic introgression from WNH fish. A small proportion of fish from Beartrap Creek was used as brood stock at the WNH until 1986, but a genetic signature of this source is absent in WNH fish. If a genetic legacy from the early use of Beartrap Creek fish as brood stock were present in contemporary WNH brood stock, straying from WNH into this creek would tend to steepen the source-sink curve and lessen a signal of introgression (Figure 5c). Even though Beartrap Creek is located a considerable distance from WNH, fish entering PWS may be attracted to Beartrap Creek, because it is a large drainage system similar to Wells River, which was the source of the WNH brood stock. Native fish spawning in Beartrap Creek also have early run timing, which is similar to that for fish returning to the WNH and Wells River.

#### Wells river

Wells River chum salmon showed the highest level of genetic introgression from hatchery salmon. This river supports the largest chum salmon population in PWS with an estimated mean run-size of 23,100 fish in the past few years (Table 6). This population shows relatively high levels of introgression despite small numbers of hatchery strays. Interacting mechanisms may explain the results for the various populations. Wells River chum salmon were the primary fish used to develop the current WNH brood stock. Hence, these hatchery fish may be more successful in spawning with Wells River fish, because of common recent ancestry. Indeed, our analysis of neutral genetic markers showed a close genetic relationship between WNH fish and the historical and contemporary samples. The two populations are also phenotypically similar, with mean egg-take at WNH occurring on July 13 (2000–2011; unpublished WNH annual reports) and with the midpoint of chum salmon escapement into Wells River on July 22 (ADF&G unpublished data). The genetic similarity measured by neutral markers and similarity in run timing may indicate that other adaptive characteristics are also similar, conferring reproductive compatibility. A constant low level of successful spawning between WNH and Wells River fish could result in the high level of introgression detected in this study.

An alternative, but not exclusive, explanation for these results is that low abundances of wild fish in a stream lead to high levels of introgression for the same intensity of straying from the hatchery. Wells River chum salmon populations were depressed during 1991–1994 and 1997–1998 with an average estimated size of only 7200 spawners. These population sizes were less than 30% of the average size of 23,089 fish (1979–2010). A proportionately larger amount of interbreeding with hatchery fish may have occurred during these years. A single successful introgression event could spread through subsequent generations, because of overlapping generations of spawning fish combined with the incorporation of hatchery genotypes into the population. As for Siwash Creek, published percentages of hatchery strays in Wells River were calculated from simple averages unweighted for overall escapement and may therefore not reflect the proportion of hatchery fish in this population. For example, although hatchery strays at Wells River averaged 7.4% over the entire spawning season (Table 6), samples during the run contained as much as 23% hatchery chum salmon (ADF&G unpublished data).

### Conclusions

The results of our study yield three important insights into detecting genetic introgression of hatchery-reared chum salmon into wild populations. First, DNA extractions from archived scales collected without using protocols for genetic sampling showed high levels of contamination with DNA from other fish. About half of the samples we attempted to use showed signs of contamination. Questionable genotypes from contaminated DNA extractions would have compromised the accuracy and power of our analyses. Rigorous quality control is an essential step in the genetic analysis of archived fish scales.

Second, the use of the source-sink model to search for temporal allele-frequency shifts appears to be a powerful method for documenting small amounts of genetic introgression over several generations. Unlike other models used for estimating genetic introgression, the source-sink model in a Bayesian context yields a confidence interval around the introgression rate. Assignments by the program STRUCTURE, which attempts to resolve population structure with the fit to Hardy-Weinberg expectations and linkage disequilibrium may not provide enough statistical power to detect small levels of introgression beyond one or two generations. Random mating is expected to lead to Hardy-Weinberg proportions in a single generation, and pseudo-linkages are expected to decay in a few generations. Small probabilities of assignment to the wrong population in our analysis likely represent type I error because of the close absolute similarity among populations. These errors were of the same order of magnitude as the probabilities of assignment to the WNH brood stock or to Wells River, the origin of the brood stock. Hence, these individual assignments may not provide evidence of introgression.

Third, the results of our study yield insights into the extent that hatchery strays have influenced wild populations of chum salmon in PWS after more than 30 years of large-scale hatchery production. These results show that some populations are more susceptible to genetic introgression by hatchery strays than other populations. Both proximity to a hatchery and the intensity of straying were less important, under some circumstances, than similarity in spawning time. Mismatches in other life-history traits may also be important in retarding genetic introgression into wild populations. Nevertheless, our results show a general convergence of allele frequencies in wild populations toward hatchery allele frequencies. While this convergence demonstrates introgression at neutral genes, the fundamental concern is over the effect of introgression on adaptive variation. Introgression from hatchery strays applies equal pressure on neutral and adaptive genes in wild populations, but it is uncertain to what extent genes underlying adaptation are resilient to introgression [50]. Future research is needed to understand extent that wild populations are adapted to a particular spawning site and the extent that the introgression of hatchery genes interrupts this adaptation.

## Supporting Information

Table S1 **Expected (** ***H*** **_E_) and observed (** ***H*** **_O_) heterozygosity for 188 single nucleotide polymorphisms in populations of chum salmon in Prince William Sound, Alaska.**
(DOCX)

Table S2 **Microsatellites used to test for contamination of chum salmon samples.**
(DOCX)

Table S3 **OpenBugs code to test for contamination between samples.**
(DOCX)

Table S4 **Pairs of single nucleotide polymorphisms (SNPs) exhibiting significant linkage disequilibrium in at least 4 of 9 collections in Prince William Sound chum salmon.**
(DOCX)

Table S5 **OpenBugs code to implement source-sink model of introgression.**
(DOCX)
