# Supplementary material for: Source-Sink Estimates of Genetic Introgression Show Influence of Hatchery Strays on Wild Chum Salmon Populations in Prince William Sound, Alaska
Source: PLoS One. 2013 Dec 13;8(12):e81916. doi: 10.1371/journal.pone.0081916 (PMC3862497; doi:10.1371/journal.pone.0081916)
Supplement: Table S1 — Expected ( H E) and observed ( H O) heterozygosity for 188 single nucleotide polymorphisms in populations of chum salmon in Prince William Sound, Alaska. (DOCX) [file pone.0081916.s001.docx]

**Table S1.** Expected (*H*_E_) and observed (*H*_O_) heterozygosity for 188 single nucleotide polymorphisms in populations of chum salmon in Prince William Sound, Alaska.

| Locus | *H*_E_ | *H*_O_ | Reference |
| --- | --- | --- | --- |
| *CTS1-627* | 0.260 | 0.245 | Elfstrom et al. (2007) |
| *Oke_ACOT-100*^3^ | 0.500 | 0.510 | Petrou et al. (in revision) |
| *Oke_AhR1-278*^5^ | 0.095 | 0.095 | Elfstrom et al. (2007) |
| *Oke_AhR1-78* | 0.496 | 0.525 | Elfstrom et al. (2007) |
| *Oke_APOB-60* | 0.461 | 0.450 | Petrou et al. (in revision) |
| *Oke_ARF* | 0.421 | 0.423 | Smith et al. (2005a) |
| *Oke_ATP5L-105*^3^ | 0.413 | 0.415 | Petrou et al. (in revision) |
| *Oke_ATP5L-248* | 0.437 | 0.431 | Petrou et al. (in revision) |
| *Oke_azin1-90* | 0.357 | 0.363 | Petrou et al. (in revision) |
| *Oke_brd2-118* | 0.240 | 0.233 | Seeb et al. (2011) |
| *Oke_brp16-65* | 0.432 | 0.433 | Petrou et al. (in revision) |
| *Oke_CATB-60* | 0.060 | 0.060 | Petrou et al. (in revision) |
| *Oke_ccd16-77* | 0.485 | 0.506 | Seeb et al. (2011) |
| *Oke_CCT3-143*^5^ | 0.085 | 0.082 | Elfstrom et al. (2007) |
| *Oke_CCT3-220* | 0.291 | 0.296 | Elfstrom et al. (2007) |
| *Oke_CD123-62* | 0.452 | 0.454 | Petrou et al. (in revision) |
| *Oke_CD81-108* | 0.032 | 0.031 | Petrou et al. (in revision) |
| *Oke_CD81-173*^3^ | 0.450 | 0.469 | Petrou et al. (in revision) |
| *Oke_cjo57-86* | 0.499 | 0.499 | Petrou et al. (in revision) |
| *Oke_CKS1-70* | 0.293 | 0.290 | Petrou et al. (in revision) |
| *Oke_CKS1-94* | 0.414 | 0.417 | Petrou et al. (in revision) |
| *Oke_CKS-389* | 0.496 | 0.504 | Smith et al. (2005b) |
| *Oke_CO1A1-72* | 0.377 | 0.389 | Petrou et al. (in revision) |
| *Oke_CO1A1-76* | 0.053 | 0.054 | Petrou et al. (in revision) |
| *Oke_col1a2-62*^3^ | 0.500 | 0.473 | Petrou et al. (in revision) |
| *Oke_Cr30^1,2^* | -- | -- | Smith et al. (2005b) |
| *Oke_Cr386^1,2^* | -- | -- | Smith et al. (2005b) |
| *Oke_ctgf-105* | 0.145 | 0.150 | Elfstrom et al. (2007) |
| *Oke_CTR2-82* | 0.495 | 0.506 | Petrou et al. (in revision) |
| *Oke_DBLOH-79* | 0.493 | 0.494 | Petrou et al. (in revision) |
| *Oke_DCXR-87* | 0.195 | 0.200 | Petrou et al. (in revision) |
| *Oke_DM20-548* | 0.498 | 0.500 | Smith et al. (2005b) |
| *Oke_e2ig5-50* | 0.498 | 0.524 | Petrou et al. (in revision) |
| *Oke_EF2-394* | 0.451 | 0.450 | Petrou et al. (in revision) |
| *Oke_EIF4EB* | 0.314 | 0.309 | Smith et al. (2005a) |
| *Oke_eif4g1-43* | 0.400 | 0.416 | Petrou et al. (in revision) |
| *Oke_f5-71* | 0.389 | 0.381 | Petrou et al. (in revision) |
| *Oke_FANK1-166* | 0.489 | 0.510 | Petrou et al. (in revision) |
| *Oke_FANK1-96*^5^ | 0.226 | 0.218 | Petrou et al. (in revision) |
| *Oke_FBXL5-61* | 0.194 | 0.194 | Petrou et al. (in revision) |
| *Oke_gdh1-191*^5^ | 0.357 | 0.364 | Petrou et al. (in revision) |
| *Oke_gdh1-234*^3^ | 0.311 | 0.313 | Petrou et al. (in revision) |
| *Oke_gdh1-62* | 0.499 | 0.504 | Petrou et al. (in revision) |
| *Oke_GHII-3129* | 0.070 | 0.069 | Elfstrom et al. (2007) |
| *Oke_glrx1-78* | 0.384 | 0.389 | Petrou et al. (in revision) |
| *Oke_GNMT-100* | 0.497 | 0.515 | Petrou et al. (in revision) |
| *Oke_GnRH-373* | 0.457 | 0.448 | Smith et al. (2005b) |
| *Oke_GPDH*^5^ | 0.254 | 0.256 | Smith et al. (2005a) |
| *Oke_GPH-105*^3^ | 0.467 | 0.468 | Elfstrom et al. (2007) |
| *Oke_GPH-78* | 0.161 | 0.162 | Elfstrom et al. (2007) |
| *Oke_H2AX-72*^3^ | 0.309 | 0.309 | Petrou et al. (in revision) |
| *Oke_hmgb1-66* | 0.467 | 0.449 | Petrou et al. (in revision) |
| *Oke_hnRNPL-239* | 0.029 | 0.025 | Elfstrom et al. (2007) |
| *Oke_HP-182* | 0.397 | 0.404 | Elfstrom et al. (2007) |
| *Oke_HSP90BA-299* | 0.051 | 0.050 | Elfstrom et al. (2007) |
| *Oke_IGFI.1* | 0.026 | 0.025 | Smith et al. (2005a) |
| *Oke_IL-1RA* | 0.180 | 0.180 | Smith et al. (2005a) |
| *Oke_IL8r2-406* | 0.335 | 0.332 | Petrou et al. (in revision) |
| *Oke_IL8r-272*^5^ | 0.176 | 0.175 | Smith et al. (2005b) |
| *Oke_KPNA2-87* | 0.079 | 0.078 | Elfstrom et al. (2007) |
| *Oke_lactb2-71* | 0.492 | 0.461 | Petrou et al. (in revision) |
| *Oke_lamp2-138*^5^ | 0.102 | 0.105 | Petrou et al. (in revision) |
| *Oke_LAMP2-186* | 0.468 | 0.472 | Petrou et al. (in revision) |
| *Oke_mcfd2-86*^3^ | 0.470 | 0.491 | Petrou et al. (in revision) |
| *Oke_METK2-97* | 0.388 | 0.402 | Petrou et al. (in revision) |
| *Oke_mgll-49* | 0.500 | 0.525 | Petrou et al. (in revision) |
| *Oke_MLRN-63* | 0.444 | 0.447 | Petrou et al. (in revision) |
| *Oke_MOESIN*^3^ | 0.234 | 0.223 | Smith et al. (2005a) |
| *Oke_nc2b-148*^3^ | 0.476 | 0.478 | Petrou et al. (in revision) |
| *Oke_ND3-69*^1,2^ | -- | -- | Smith et al. (2005b) |
| *Oke_ndub3-58* | 0.072 | 0.055 | Petrou et al. (in revision) |
| *Oke_NHERF-123*^3^ | 0.343 | 0.308 | Petrou et al. (in revision) |
| *Oke_NHERF-54* | 0.312 | 0.297 | Petrou et al. (in revision) |
| *Oke_NUPR1-70* | 0.329 | 0.327 | Petrou et al. (in revision) |
| *Oke_PDIA3-475*^3^ | 0.385 | 0.397 | Petrou et al. (in revision) |
| *Oke_PDIA3-82* | 0.439 | 0.442 | Petrou et al. (in revision) |
| *Oke_pgap-111* | 0.438 | 0.444 | Petrou et al. (in revision) |
| *Oke_pgap-92*^5^ | 0.371 | 0.381 | Petrou et al. (in revision) |
| *Oke_pnrc2-78* | 0.354 | 0.339 | Petrou et al. (in revision) |
| *Oke_psmd9-188* | 0.451 | 0.460 | Petrou et al. (in revision) |
| *Oke_psmd9-57*^5^ | 0.159 | 0.157 | Petrou et al. (in revision) |
| *Oke_rab5a-117* | 0.453 | 0.454 | Petrou et al. (in revision) |
| *Oke_ras1-362*^3^ | 0.486 | 0.500 | Elfstrom et al. (2007) |
| *Oke_RFC2* | 0.039 | 0.036 | Smith et al. (2005a) |
| *Oke_RH1OP* | 0.473 | 0.492 | Smith et al. (2005a) |
| *Oke_ROA1-209* | 0.393 | 0.375 | Petrou et al. (in revision) |
| *Oke_RPN1-80*^3^ | 0.496 | 0.543 | Petrou et al. (in revision) |
| *Oke_RS27-81* | 0.315 | 0.319 | Petrou et al. (in revision) |
| *Oke_RS27-94*^3^ | 0.283 | 0.197 | Petrou et al. (in revision) |
| *Oke_RS9-379*^3^ | 0.497 | 0.464 | Petrou et al. (in revision) |
| *Oke_RSPRY1-106* | 0.107 | 0.104 | Seeb et al. (2011) |
| *Oke_serpin* | 0.494 | 0.496 | Smith et al. (2005a) |
| *Oke_slc1a3a-86* | 0.482 | 0.469 | Petrou et al. (in revision) |
| *Oke_sylc-90* | 0.371 | 0.363 | Petrou et al. (in revision) |
| *Oke_TCP1-78* | 0.163 | 0.158 | Elfstrom et al. (2007) |
| *Oke_TCTA-202* | 0.490 | 0.494 | Petrou et al. (in revision) |
| *Oke_TCTA-99*^5^ | 0.435 | 0.461 | Petrou et al. (in revision) |
| *Oke_Tf-278* | 0.500 | 0.482 | Elfstrom et al. (2007) |
| *Oke_thic-84* | 0.492 | 0.474 | Petrou et al. (in revision) |
| *Oke_txnrd1-74* | 0.360 | 0.349 | Petrou et al. (in revision) |
| *Oke_u0602-244*^3^ | 0.493 | 0.501 | Seeb et al. (2011) |
| *Oke_U1001-79*^5^ | 0.495 | 0.514 | Seeb et al. (2011) |
| *Oke_U1002-165*^5^ | 0.410 | 0.401 | Seeb et al. (2011) |
| *Oke_U1002-262* | 0.496 | 0.478 | Seeb et al. (2011) |
| *Oke_U1008-83* | 0.173 | 0.169 | Seeb et al. (2011) |
| *Oke_U1010-154*^2^ | 0.000 | 0.000 | Seeb et al. (2011) |
| *Oke_U1010-251*^3^ | 0.486 | 0.485 | Seeb et al. (2011) |
| *Oke_U1012-241* | 0.500 | 0.510 | Seeb et al. (2011) |
| *Oke_U1012-60*^5^ | 0.500 | 0.501 | Seeb et al. (2011) |
| *Oke_U1015-255* | 0.496 | 0.472 | Seeb et al. (2011) |
| *Oke_U1016-154*^4^ | 0.493 | 0.545 | Seeb et al. (2011) |
| *Oke_U1017-52* | 0.310 | 0.299 | Seeb et al. (2011) |
| *Oke_U1018-50* | 0.009 | 0.009 | Seeb et al. (2011) |
| *Oke_U1019-218* | 0.006 | 0.006 | Seeb et al. (2011) |
| *Oke_U1020-75*^3^ | 0.341 | 0.370 | Seeb et al. (2011) |
| *Oke_U1021-102* | 0.350 | 0.343 | Seeb et al. (2011) |
| *Oke_U1022-114*^5^ | 0.263 | 0.255 | Seeb et al. (2011) |
| *Oke_U1022-139*^5^ | 0.312 | 0.311 | Seeb et al. (2011) |
| *Oke_U1023-147* | 0.486 | 0.524 | Seeb et al. (2011) |
| *Oke_U1024-113* | 0.030 | 0.029 | Seeb et al. (2011) |
| *Oke_U1025-135* | 0.033 | 0.032 | Seeb et al. (2011) |
| *Oke_U1027-89*^3^ | 0.394 | 0.345 | Seeb et al. (2011) |
| *Oke_U1028-100*^3^ | 0.422 | 0.435 | Seeb et al. (2011) |
| *Oke_U1031-132* | 0.154 | 0.151 | Seeb et al. (2011) |
| *Oke_U1103-150*^3^ | 0.424 | 0.370 | Petrou et al. (in revision) |
| *Oke_u1-519* | 0.482 | 0.494 | Smith et al. (2005b) |
| *Oke_U2001-629*^3^ | 0.485 | 0.473 | Petrou et al. (in revision) |
| *Oke_U2002-200* | 0.493 | 0.494 | Petrou et al. (in revision) |
| *Oke_U2003-142* | 0.027 | 0.027 | Petrou et al. (in revision) |
| *Oke_U2005-62* | 0.411 | 0.405 | Petrou et al. (in revision) |
| *Oke_U2006-109* | 0.484 | 0.492 | Petrou et al. (in revision) |
| *Oke_U2007-190* | 0.466 | 0.450 | Petrou et al. (in revision) |
| *Oke_U2010-94* | 0.496 | 0.497 | Petrou et al. (in revision) |
| *Oke_U2011-107* | 0.077 | 0.077 | Petrou et al. (in revision) |
| *Oke_U2015-151* | 0.119 | 0.121 | Petrou et al. (in revision) |
| *Oke_U2016-118* | 0.369 | 0.371 | Petrou et al. (in revision) |
| *Oke_U2017-87* | 0.083 | 0.087 | Petrou et al. (in revision) |
| *Oke_U2019-112* | 0.336 | 0.336 | Petrou et al. (in revision) |
| *Oke_U202^2^* | 0.001 | 0.001 | Smith et al. (2005a) |
| *Oke_U2020-51* | 0.410 | 0.395 | Petrou et al. (in revision) |
| *Oke_U2021-86* | 0.499 | 0.492 | Petrou et al. (in revision) |
| *Oke_U2022-101* | 0.120 | 0.115 | Petrou et al. (in revision) |
| *Oke_U2023-99* | 0.057 | 0.058 | Petrou et al. (in revision) |
| *Oke_U2024-93*^3^ | 0.424 | 0.425 | Petrou et al. (in revision) |
| *Oke_U2025-86* | 0.499 | 0.443 | Petrou et al. (in revision) |
| *Oke_U2026-64* | 0.474 | 0.465 | Petrou et al. (in revision) |
| *Oke_U2029-79* | 0.500 | 0.506 | Petrou et al. (in revision) |
| *Oke_U2031-37* | 0.105 | 0.104 | Petrou et al. (in revision) |
| *Oke_U2032-74*^3^ | 0.230 | 0.224 | Petrou et al. (in revision) |
| *Oke_U2033-122* | 0.333 | 0.338 | Petrou et al. (in revision) |
| *Oke_U2034-55* | 0.460 | 0.452 | Petrou et al. (in revision) |
| *Oke_U2035-54* | 0.102 | 0.103 | Petrou et al. (in revision) |
| *Oke_U2037-76* | 0.156 | 0.159 | Petrou et al. (in revision) |
| *Oke_U2038-32* | 0.099 | 0.098 | Petrou et al. (in revision) |
| *Oke_U2040-77* | 0.478 | 0.450 | Petrou et al. (in revision) |
| *Oke_U2041-84* | 0.499 | 0.484 | Petrou et al. (in revision) |
| *Oke_U2042-61* | 0.295 | 0.294 | Petrou et al. (in revision) |
| *Oke_U2043-51* | 0.160 | 0.164 | Petrou et al. (in revision) |
| *Oke_U2045-43* | 0.409 | 0.414 | Petrou et al. (in revision) |
| *Oke_U2047-49* | 0.287 | 0.282 | Petrou et al. (in revision) |
| *Oke_U2048-91* | 0.343 | 0.353 | Petrou et al. (in revision) |
| *Oke_U2049-99* | 0.446 | 0.460 | Petrou et al. (in revision) |
| *Oke_U2050-101* | 0.178 | 0.180 | Petrou et al. (in revision) |
| *Oke_U2052-56* | 0.320 | 0.325 | Petrou et al. (in revision) |
| *Oke_U2053-60*^3^ | 0.433 | 0.414 | Petrou et al. (in revision) |
| *Oke_U2054-58* | 0.125 | 0.124 | Petrou et al. (in revision) |
| *Oke_U2056-90*^3^ | 0.500 | 0.506 | Petrou et al. (in revision) |
| *Oke_U2057-80* | 0.480 | 0.482 | Petrou et al. (in revision) |
| *Oke_U212* | 0.101 | 0.098 | Smith et al. (2005a) |
| *Oke_U216*^3^ | 0.136 | 0.129 | Smith et al. (2005a) |
| *Oke_U217* | 0.397 | 0.392 | Smith et al. (2005a) |
| *Oke_U22* | 0.492 | 0.473 | Smith et al. (2005a) |
| *Oke_U302-195* | 0.042 | 0.035 | Elfstrom et al. (2007) |
| *Oke_U502-241* | 0.282 | 0.281 | Elfstrom et al. (2007) |
| *Oke_U503-272* | 0.033 | 0.033 | Elfstrom et al. (2007) |
| *Oke_U504-228*^3^ | 0.284 | 0.300 | Elfstrom et al. (2007) |
| *Oke_U505-112*^3^ | 0.450 | 0.465 | Elfstrom et al. (2007) |
| *Oke_U506-110* | 0.492 | 0.510 | Elfstrom et al. (2007) |
| *Oke_U507-286* | 0.499 | 0.515 | Elfstrom et al. (2007) |
| *Oke_U507-87*^5^ | 0.419 | 0.411 | Elfstrom et al. (2007) |
| *Oke_U509-219* | 0.452 | 0.475 | Elfstrom et al. (2007) |
| *Oke_U510-204* | 0.374 | 0.369 | Elfstrom et al. (2007) |
| *Oke_U511-271* | 0.095 | 0.094 | Elfstrom et al. (2007) |
| *Oke_U514-150* | 0.052 | 0.050 | Elfstrom et al. (2007) |
| *Oke_UBA3-245*^3^ | 0.489 | 0.507 | Seeb et al. (2011) |
| *Oke_uqcrfs-69* | 0.010 | 0.010 | Petrou et al. (in revision) |
| *Oke_XBP1-82* | 0.412 | 0.410 | Petrou et al. (in revision) |
| *Oke_zn593-152* | 0.384 | 0.393 | Petrou et al. (in revision) |
|  |  |  |  |

^1^ SNPs from mtDNA loci.

^2^ SNPs were dropped because they were monomorphic.

^3^ SNPs were eliminated because of poor success rate of amplification on historical scale collections.

^4^ SNPs were eliminated due to failing Hardy-Weinberg expectations.

^5^ SNPs dropped due to linkage.

References

Elfstrom CM, Smith CT, Seeb LW (2007) Thirty-eight single nucleotide polymorphism markers for high-throughput genotyping of chum salmon. Molecular Ecology Notes 7: 1211–1215.

Petrou EL, Hauser L, Waples RS, Seeb JE, Templin WD, Gomez-Uchida D, Seeb LW (in revision) Secondary contact and changes in coastal hydrology influence the nonequilibrium population structure of a salmonid (*Oncorhynchus keta*). Molecular Ecology Resources.

Seeb, JE, Pascal CE, Grau ED, Seeb LW, Templin WD, Harkins T, Roberts SB. (2011) Transcriptome sequencing and high-resolution melt analysis advance single nucleotide polymorphism discovery in duplicated salmonids. Molecular Ecology Resources 11: 335–348.

Smith CT, Elfstrom CM, Seeb JE, Seeb LW (2005a) Use of sequence data from rainbow trout and Atlantic salmon for SNP detection in Pacific salmon. Molecular Ecology 14: 4193–4203.

Smith CT, Baker J, Park L, Seeb LW, Elfstrom, et al. (2005b) Characterization of 13 single nucleotide polymorphism markers for chum salmon. Molecular Ecology Notes 5: 259–262.
