# Supplementary material for: Source-Sink Estimates of Genetic Introgression Show Influence of Hatchery Strays on Wild Chum Salmon Populations in Prince William Sound, Alaska
Source: PLoS One. 2013 Dec 13;8(12):e81916. doi: 10.1371/journal.pone.0081916 (PMC3862497; doi:10.1371/journal.pone.0081916)
Supplement: Table S2 — Microsatellites used to test for contamination of chum salmon samples. (DOCX) [file pone.0081916.s002.docx]

**Table S2.** Microsatellites used to test for contamination of chum salmon samples.

| Locus | Reference |
| --- | --- |
| Oke4 | Buchholz et al. 2001 |
| Oke11 | Buchholz et al. 2001 |
| Oki1L | Smith et al. 1998 |
| Oki1U | Smith et al. 1998 |
| Ots2.1L | Banks et al. 1999 |
| Ots2.1U | Banks et al. 1999 |
| Ots103 | Small et al. 1998 |

Amplification conditions: PCR was conducted in 10 µL volumes containing 1.5 µL of undiluted PCR product, 0.25 µM of Oke4 (PET™), Oke11 (PET™), Oki1 (6FAM™), Ots2.1 (NED™), and Ots103 (VIC®) forward and reverse primers, 2 mM MgCl2, 0.2 mM each dNTPs, 0.5 units GoTaq® Flexi DNA Polymerase (Promega) and Promega Proprietary 5x™ Colorless GoTaq® Flexi Buffer (pH 8.5). Markers were amplified with a Applied Biosystems 9700 thermalcycler using a PCR protocol of 95°C (2min) followed by 20 cycles of: 92 °C (30 s), 58 °C (1 min), 72 °C (1 min) and a final extension at 72 °C (1 min). PCR products were size fractionated on an ABI3730 DNA Analyzer with a GeneScan™ 500 LIZ™ size standard and analyzed using the local Southern sizing algorithm in the GeneMapper® v4.1 software (all Applied Biosystems, Inc.). The primer pair Oki1 amplifies PCR product for markers Oki1L and Oki1u. Also, the primer pair Ots2.1 amplifies PCR product for markers Ots2.1L and Ots2.1u. Contaminated samples were identified as those with more than two alleles present at any of the 7 markers. Contaminated samples were included in the SNP genotyping assays to explore the influence that contamination had on allelic discrimination, but they were removed prior to final SNP genotyping and from all further analysis.

References

Banks M A, Blouin MS, Baldwin BA, Rashbrook VK, Fitzgerald HA, Blankenship SM, Hedgecock D (1999). Isolation and inheritance of novel microsatellites in chinook salmon (*Oncorhynchus tshawytscha*). Journal of Heredity 90: 281–288.

Buchholz W, Miller SJ, Spearman WJ (2001) Isolation and characterization of chum salmon microsatellite loci and use across species. Animal Genetics 32: 160–167.

Small MP, Beacham TD, Withler RE, Nelson RJ (1998) Discriminating coho salmon (*Oncorhynchus kisutch*) populations within the Fraser River, British Columbia, using microsatellite DNA markers. Molecular Ecology 7: 141–155.

Small MP, Beacham TD, Withler RE, Nelson RJ (1998) Discriminating coho salmon (*Oncorhynchus kisutch*) populations within the Fraser River, British Columbia, using microsatellite DNA markers. Molecular Ecology 7: 141–155.

Smith CT, Koop BF, Nelson RJ (1998) Isolation and characterization of coho salmon (*Oncorhynchus kisutch*) microsatellites and their use in other salmonids. Molecular Ecology 11: 1614–1616.
