# Supplementary material for: Source-Sink Estimates of Genetic Introgression Show Influence of Hatchery Strays on Wild Chum Salmon Populations in Prince William Sound, Alaska
Source: PLoS One. 2013 Dec 13;8(12):e81916. doi: 10.1371/journal.pone.0081916 (PMC3862497; doi:10.1371/journal.pone.0081916)
Supplement: Table S3 — OpenBugs code to test for contamination between samples. (DOCX) [file pone.0081916.s003.docx]

**Table S3.** OpenBugs code to test for contamination between samples.

model{

for(i in 1:C){ # C=number collections

for(k in 1:N[i]){ # N[i] = number inividuals in collection i

z[i,k] ~ dbern(0.5) # z[i,k] = contamination indicator for individual k

for(*l* in 1:L){ # L = number loci

x[i,k,*l*,1:3] ~ dmulti(P[i,k,*l*,1:3],1) # Likelihood

for(j in 1:3){ # P[i,k,*l*,j] = individual k's genotype frequency j

P[i,k,*l*,j] <- pow(p1[i,*l*,j],z[i,k])*pow(p0[i,*l*,j],1-z[i,k])

}

}

}

for(*l* in 1:L){

pi[i,*l* ] ~ dbeta(0.5,0.5) # allele frequency = Prob(A)

p0[i,*l* ,1] <- pow(q[i,*l*],2) # uncontaminated genotype 1

p0[i,*l* ,2] <- 2*q[i,*l*]*(1-q[i,*l*]) # uncontaminated genotype 2

p0[i,*l* ,3] <- pow(1-q[i,*l*],2) # uncontaminated genotype 3

p1[i,*l* ,1] <- pow(q[i,*l*],4) # contaminated genotype 1

p1[i,*l* ,2] <- 1-p1[i,*l*,1]-p1[i,*l*,3] # contaminated genotype 2

p1[i,*l* ,3] <- pow(1-q[i,*l*],4) # contaminated genotype 3

}

}

}
