# Supplementary material for: Source-Sink Estimates of Genetic Introgression Show Influence of Hatchery Strays on Wild Chum Salmon Populations in Prince William Sound, Alaska
Source: PLoS One. 2013 Dec 13;8(12):e81916. doi: 10.1371/journal.pone.0081916 (PMC3862497; doi:10.1371/journal.pone.0081916)
Supplement: Table S4 — Pairs of single nucleotide polymorphisms (SNPs) exhibiting significant linkage disequilibrium in at least 4 of 9 collections in Prince William Sound chum salmon. (DOCX) [file pone.0081916.s004.docx]

Table S4. Pairs of single nucleotide polymorphisms (SNPs) exhibiting significant linkage disequilibrium in at least 4 of 9 collections in Prince William Sound chum salmon. The decision to retain a locus was based on the percentage of historical fish missing genotype data for each locus and the observed heterozygosity for all collections combined.

| Pair | Locus | % missing  (historical) | *H*_O_ (All) | No. pops  in LD | Decision |
| --- | --- | --- | --- | --- | --- |
| 1 | *Oke_DBLOH-79* | 5.0 | 0.494 | 9 | Retain |
| 1 | *Oke_U1001-79* | 7.1 | 0.496 | 9 | Discard |
| 2 | *Oke_U1022-114* | 10.5 | 0.254 | 9,6 | Discard |
| 2 | *Oke_U1021-102* | 6.3 | 0.343 | 6,6 | Retain |
| 2 | *Oke_U1022-139* | 4.6 | 0.300 | 9,6 | Discard |
| 3 | *Oke_IL8r2-406* | 6.3 | 0.332 | 9 | Retain |
| 3 | *Oke_IL8r-272* | 2.5 | 0.182 | 9 | Discard |
| 4 | *Oke_TCTA-202* | 6.7 | 0.494 | 9 | Retain |
| 4 | *Oke_TCTA-99* | 6.3 | 0.446 | 9 | Discard |
| 5 | *Oke_U1012-241* | 8.8 | 0.510 | 9 | Retain |
| 5 | *Oke_U1012-60* | 10.0 | 0.501 | 9 | Discard |
| 6 | *Oke_U507-286* | 7.1 | 0.515 | 9 | Retain |
| 6 | *Oke_U507-87* | 8.4 | 0.397 | 9 | Discard |
| 7 | *Oke_CCT3-143* | 2.1 | 0.082 | 8 | Discard |
| 7 | *Oke_CCT3-220* | 4.2 | 0.296 | 8 | Retain |
| 8 | *Oke_FANK1-96* | 5.9 | 0.205 | 8 | Discard |
| 8 | *Oke_FANK1-166* | 3.8 | 0.510 | 8 | Retain |
| 9 | *Oke_gdh1-191* | 3.8 | 0.361 | 8 | Discard |
| 9 | *Oke_gdh1-62* | 7.5 | 0.504 | 8 | Retain |
| 10 | *Oke_pgap-111* | 5.0 | 0.444 | 8 | Retain |
| 10 | *Oke_pgap-92* | 9.2 | 0.379 | 8 | Discard |
| 11 | *Oke_LAMP2-186* | 6.3 | 0.472 | 6 | Retain |
| 11 | *Oke_lamp2-138* | 2.1 | 0.104 | 6 | Discard |
| 12 | *Oke_U1002-262* | 4.6 | 0.478 | 6 | Retain |
| 12 | *Oke_U1002-165* | 9.2 | 0.390 | 6 | Discard |
| 13 | *Oke_psmd9-188* | 6.7 | 0.460 | 4 | Retain |
| 13 | *Oke_psmd9-57* | 2.1 | 0.158 | 4 | Discard |
| 14 | *Oke_AhR1-78* | 6.7 | 0.525 | 5 | Retain |
| 14 | *Oke_AhR1-278* | 2.1 | 0.096 | 5 | Discard |
| 15 | *Oke_GPDH* | 3.8 | 0.250 | 5 | Discard |
| 15 | *Oke_zn593-152* | 8.4 | 0.393 | 5 | Retain |
