# Supplementary material for: Source-Sink Estimates of Genetic Introgression Show Influence of Hatchery Strays on Wild Chum Salmon Populations in Prince William Sound, Alaska
Source: PLoS One. 2013 Dec 13;8(12):e81916. doi: 10.1371/journal.pone.0081916 (PMC3862497; doi:10.1371/journal.pone.0081916)
Supplement: Table S5 — OpenBugs code to implement source-sink model of introgression. (DOCX) [file pone.0081916.s005.docx]

Table S5. OpenBugs code to implement source-sink model of introgression.

model{

for(*l* in 1:L){

y_Source[*l*] ~ dbin(q_Source[*l*],N_Source[*l*])

q_Source[*l*] ~ dbeta(0.5,0.5)

for(i in 1:C){

y0_Sink[i,*l*] ~ dbin(q0_Sink[i,*l*],n0_Sink[i,*l*])

yn_Sink[i,*l*] ~ dbin(qn_Sink[i,*l*],Nn_Sink[i,*l*])

q0_Sink[i,*l*] ~ dbeta(0.5,0.5)

qn_Sink [i,*l*] <- mprime[i]*(q0_Sink[i,*l*]-q_Source[*l*])+q_Source[*l*]

}

}

for(i in 1:C){

mprime[i] ~ dnorm(0,0.001)

}

}
